# Kv4.3 expression reverses I_Ca_ remodeling in ventricular myocytes of heart failure

**DOI:** 10.18632/oncotarget.21956

**Published:** 2017-10-23

**Authors:** Jun Cheng, Jianlei Cao, Xingchen Jiang, Lin Xu, Yanggan Wang

**Affiliations:** ^1^ Department of Cardiology, Zhongnan Hospital of Wuhan University, Wuhan University, Wuhan 430071, China; ^2^ Medical Research Institute of Wuhan University, Wuhan University, Wuhan 430071, China; ^3^ Department of Cardiology, Renmin Hospital of Wuhan University, Wuhan University, Wuhan 430060, China

**Keywords:** L-type calcium current, Kv4.3, CaMKII, myocytes, heart failure

## Abstract

**Background:**

Ca^2+^/calmodulin-dependent protein kinase II (CaMKII)-dependent L-type calcium channel (LTCC) current (I_Ca_) remodeling is an important contributor to the disruption of calcium homeostasis in heart failure (HF). We have reported that Kv4.3 proteins play an important role in delicate regulation of the membrane-associated CaMKII activity in ventricular myocytes. Here, we investigated the effect of *in vivo* Kv4.3 expression on I_Ca_ in HF left ventricular (LV) myocytes.

**Results:**

Kv4.3 expression reduced overall CaMKII autophosphorylation with much greater reduction in the membrane compartmentalized CaMKII activity. I_Ca_ density in subepicardial (SEP) and subendocardial (SEN) myocytes was proportionately reduced, without changing the transmural gradient. While the time course of I_Ca_ decay was hastened, the voltage-dependence of I_Ca_ activation and inactivation, however, remained unchanged. I_Ca_ recovery from inactivation was significantly accelerated. In line with the partial inhibition of CaMKII, the frequency-dependent Ca^2+^-induced I_Ca_ facilitation was recovered in the HF myocytes transfected with Kv4.3.

**Materials and Methods:**

Pressure-overload HF was induced by thoracic aortic banding. Kv4.3 expression was achieved by Ad-Kv4.3 injection in the LV myocardium. I_Ca_ was recorded in dissociated SEP and SEN myocytes using whole-cell patch clamp method.

**Conclusions:**

Kv4.3 expression in HF ventricle can effectively reverse I_Ca_ remodeling via inhibition of the membrane-associated CaMKII, pointing to Kv4.3 restoration as a potential therapeutic approach for the disordered calcium regulation in HF.

## INTRODUCTION

I_Ca_ is the key mediator for the excitation-contraction (E-C) coupling in cardiomyocytes. Ca^2+^ entry through L-type calcium channel (LTCC) triggers a release of Ca^2+^ from sarcoplasmic reticulum (SR), initiating myofilament contraction. The amplitude and the kinetics of I_Ca_ determine the amount of Ca^2+^ entry and consequently the contractile force and relaxation. In addition, Ca^2+^ entry and SR Ca^2+^ release activates a number of Ca^2+^-sensitive signaling cascades, most important among them is Ca^2+^/calmodulin-dependent protein kinase II (CaMKII). CaMKII plays a key role in regulation of E-C coupling by phosphorylation of a number of proteins involved in Ca^2+^ handling, such as LTCC, ryanodine receptor (RyR2), sarco/endoplasmic reticulum Ca^2+^-ATPase (SERCA) and phospholamban (PLB) [1]. It is also a known powerful stimulator of the excitation-transcription (E-T) coupling mediated by class II histone deacetylases and myocyte enhancer factor 2 [2] and a arrhythmogenic molecule by regulation of several important membrane ion channels [3], of which the I_Ca_, transient outward current (I_to_) and late sodium current (I_NaL_) are most extensively studied [4, 5]. In myocytes, CaMKII activation is initiated by Ca^2+^/CaM binding and can be mediated by β-AR agonist stimulation and oxidative stress. In the setting of heart failure (HF), reduction of I_to_ and increase of I_NaL_ prolong action potential duration (APD) which increases Ca^2+^ entry through LTCC and the subsequent SR Ca^2+^ release, leading to Ca^2+^ overload and CaMKII activation, and this process is facilitated by the enhanced β-AR adrenergic stimulation and oxidative stress, which causes excessive CaMKII activation. On the other hand, increased CaMKII activity hyperphosphorylates LTCC, leading to an increase in I_Ca_ magnitude and slowing of the inactivation time course. This induces a consequent increase in Ca^2+^ entry and further CaMKII activation. In other words, HF-related I_Ca_ remodeling is a CaMKII-dependent and self-exacerbated process. Indeed, we have recently reported that HF-related I_Ca_ remodeling is a result of increased CaMKII-dependent Ca^2+^ channel phosphorylation, whereas other regulatory pathways, *e.g.* PKA, may only play a minor role (if any) [6].

In fact, in the normal heart, a beat-to-beat CaMKII activation is delicately regulated, which effectively prevents CaMKII from over-activation. For instant, we have recently reported an important mechanism which can prevent calcium-induced CaMKII hyperphosphorylation [7]. We demonstrated that a significant amount of inactive CaMKII in myocytes is preserved in the CaMKII-Kv4.3 molecular complex. The Kv4.3-coupled CaMKII cannot be activated by elevated [Ca^2+^]_i_. This finding revealed that the CaMKII-Kv4.3 units are important intrinsic CaMKII inhibitor for the delicate regulation of CaMKII activity in ventricular myocytes. However, a growing body of evidence has elucidated that a reduction of I_to_ is a well-known feature of ventricular myocytes from HF animal models and patients, and this remodeling occurs at the early developing stage [8, 9]. Importantly, the majority of experiments in animal models and HF patients have correlated the decrease in I_to_ with the reduction of Kv4.3 expression [8, 10, 11], implicating Kv4.3 down-regulation in excessive CaMKII activation and the consequent remodeling of cellular Ca^2+^ handling in HF. In the current study, we tested whether Kv4.3 expression (restoration) can reverse I_Ca_ remodeling in HF ventricular myocytes, which is known mainly caused by excessive CaMKII activation.

## RESULTS

### Kv4.3 transfection in HF ventricular myocytes

**Figure 1:**
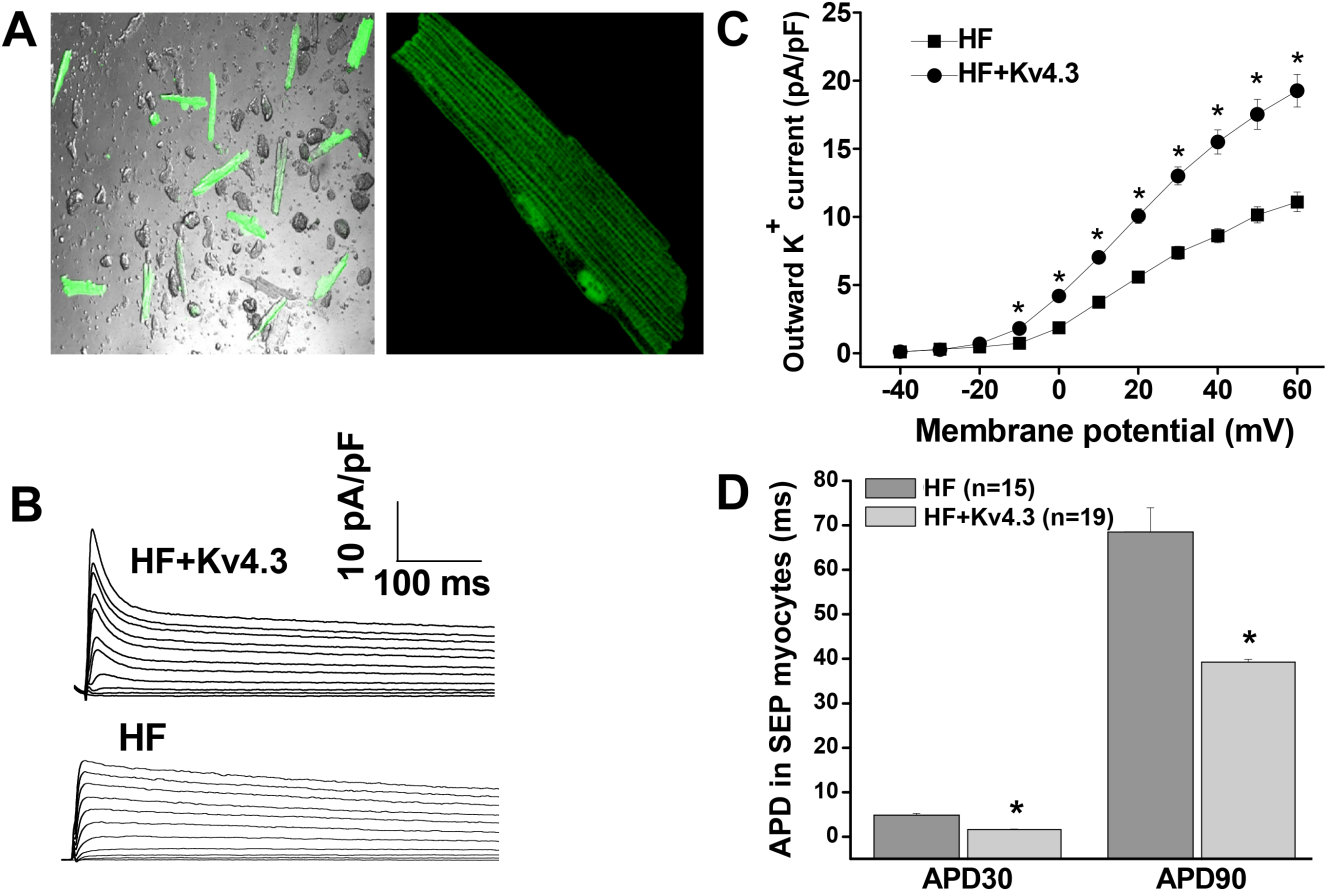
*In vivo* adenovirus-mediated transfection of Kv4.3 in HF ventricular myocytes **(Panel A)** Isolated LV myocytes from HF mice 7 days after Ad-Kv4.3 transfection (n=5). Green cells indicate successful Kv4.3 expression. **(Panel B)** Representative current traces of transient outward current elicited by 5000 ms test pulses in 10 mV increments from a holding potential of -80 mV to +60 mV at pulse intervals of 10 s. **(Panel C)** Voltage relationship of transient outward current recorded in HF myocytes with (n=10) and without Ad-Kv4.3 (n=10) transfection, respectively. Current amplitudes were normalized to cell capacitance and plotted as mean values. **(Panel D)** Action potential durations recorded in the isolated LV subepicardial myocytes with and without Ad-Kv4.3 transfection. Vertical bars represent S.E.M. * denotes *p* < 0.05, compared to the controls.

HF was successfully produced 2 weeks after sTAB in mice confirmed by echocardiogram, with an increase in LV end-diastolic volume (from 38.2 ± 0.7 to 90.5 ± 3.0 μl) and decrease in ejection fraction (from 83.1 ± 0.5% to 38.8 ± 1.2%) (p <0.05, n=31). Ad-Kv4.3 was then injected to the LV myocardium in the HF mice. 7 days after *in vivo* adenovirus injection, HF mice transfected with Ad-Kv4.3 (n = 10) was used for myocyte isolation. Consistent with our previous results [7], multisite Ad-v4.3 injection in adult mouse LV produced an overall transfection rate of 70%, and the transfected myocytes showed normal shape and clear striations (Figure 1A & 1B). As a limit of multisite Ad-v4.3 injection, Kv4.3 transfection was heterogeneously distributed in the LV wall, with a higher transfection rate in the endocardium than epicardium [7]. Transient outward current was recorded in HF ventricular myocytes with and without Ad-Kv4.3 expression, respectively. The GFP positive myocytes under the fluorescence microscope indicate successful Kv4.3 expression. As expected, myocytes transfected with Ad-K4.3 manifested a nearly 2-fold increase in the transient outward current density, indicating successful expression of Kv4.3 channel (Figure 1B & 1C). The transient outward current density at +60 mV was increased from 11.09 ± 0.72 A/pF to 19.26 ± 1.19 pA/pF, a level similar to the that recorded in the endocardial myocytes isolated from the normal mice (19.2±1.1 pA/pF) [12]. In line with this, action potentials recorded in the Kv4.3 transfected myocytes manifested significant shortening of APD (Figure 1D).

### Inhibition of CaMKII activity by Kv4.3 expression in HF ventricular myocytes

**Figure 2:**
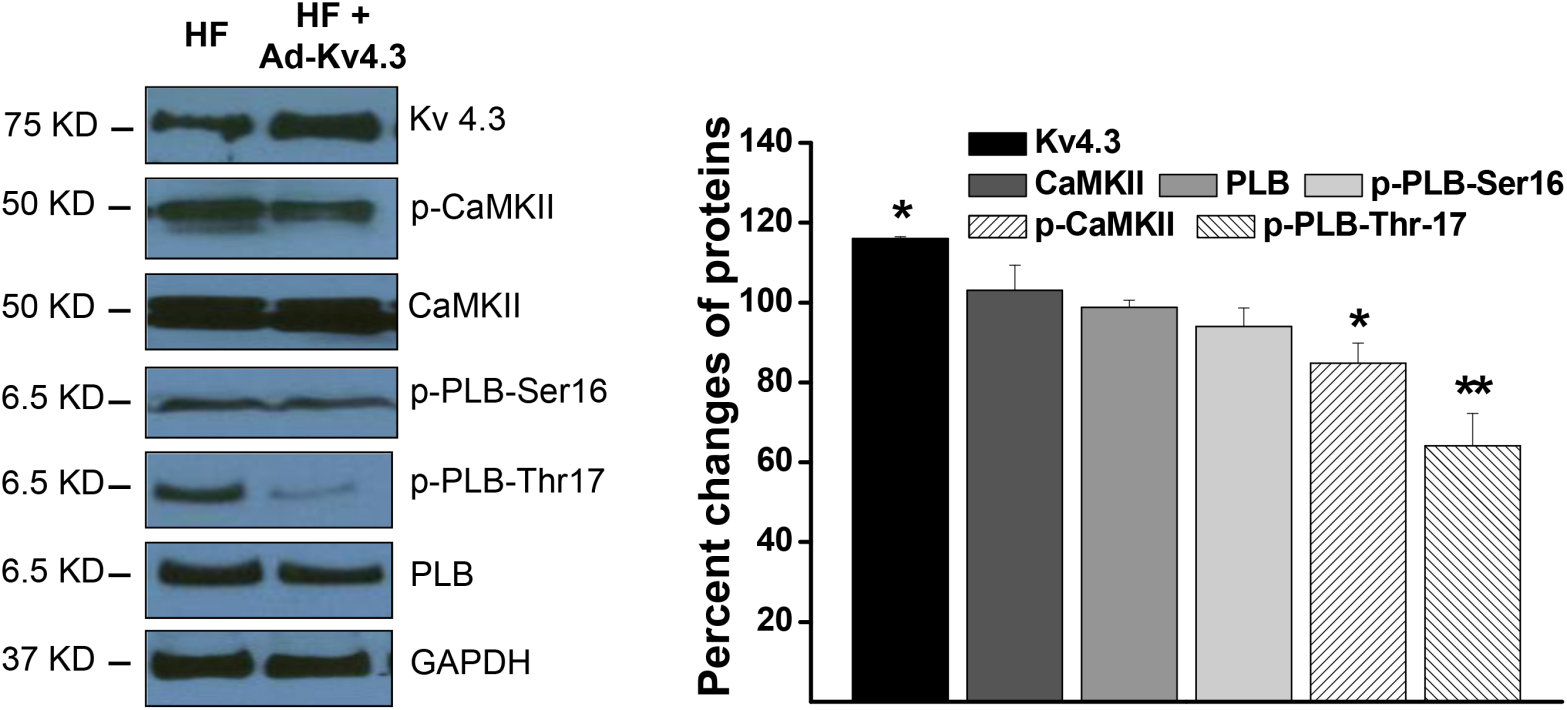
Western blot results showing protein expressions in the HF LV myocytes with and without Ad-Kv4.3 transfection, respectively The left panel shows representative examples of Western blots. The statistic values shown in the right panel were from 3 mice and 3 independent experiments for each protein. * denotes *p* < 0.05, ** denotes *p* < 0.01, compared to the controls.

We have recently demonstrated that *in vivo* Kv4.3 transfection in LV ventricular myocytes in the normal mice significantly inhibited CaMKII activity by direct binding to the CaM binding sites and forming a CaMKII-Kv4.3 molecular complex [7]. Here, we tested the effect of Kv4.3 transfection on CaMKII inhibition in HF ventricular myocytes. We found that in the Ad-Kv4.3 transfected HF LV, CaMKII autophosphorylation was significantly reduced with a much larger reduction in the compartmentalized CaMKII activity in the SR region which was manifested by a much greater inhibition of the PLB phosphorylation at Thr17, a CaMKII-dependent phosphorylation site (Figure 2). These results suggest that Kv4.3 expression in failing ventricle caused a predominant inhibition of the membrane compartmentalized CaMKII activity, with only minor inhibition of the overall intracellular CaMKII activity. This is consistent with the membrane distribution of CaMKII-Kv4.3 units, *i.e.* CaMKII-Kv4.3 units protect against activation of CaMKII in the sarcolemmal membrane microdomains by trapping inactive CaMKII to CaMKII-Kv4.3 units and preventing it from being activated by elevated Ca^2+^ in the microdomain (*e.g*. LTCC-SR microdomain [13]).

### Kv4.3 expression in HF ventricular myocytes reversed I_Ca_ density and inactivation time course

**Figure 3:**
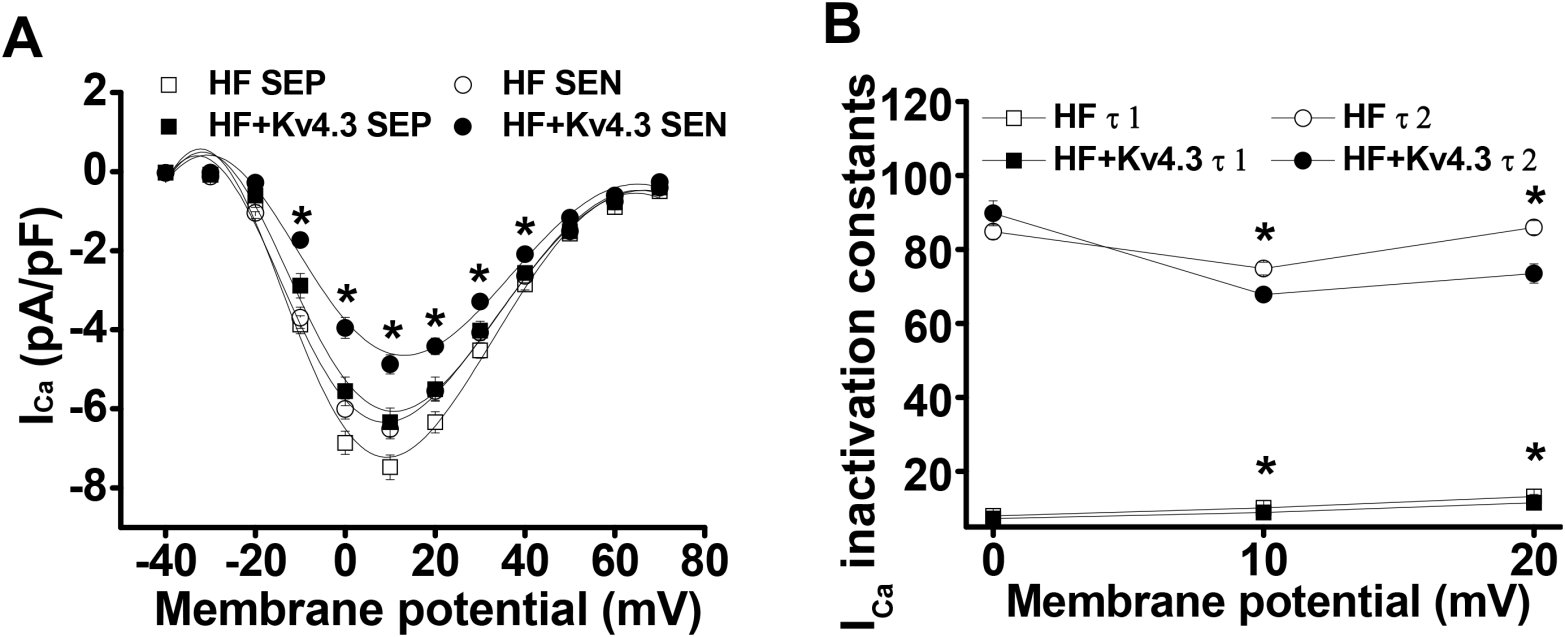
**(Panel A)** I_Ca_**-**voltage relationship recorded in the SEP and SEN myocytes from the HF LV with and without Ad-Kv4.3 transfection, respectively. Current densities were plotted as mean values. Vertical bars represent S.E.M. Peak I_Ca_ (recorded at test potential of +10 mV) was reduced from 7.48 ± 0.31 pA/pF (n=23) to 6.34 ± 0.35 pA/pF (n=15) for SEP and from 6.51 ± 0.25 pA/pF (n=23) to 4.9 ± 0.24 pA/pF (n=15) for SEN cells, respectively. **(Panel B)** Statistic analysis of I_Ca_ inactivation time constants recorded from the HF LV myocytes with (n=36) and without Ad-Kv4.3 (n=46) transfection, respectively. The decay phase was fit by biexponential function. Mean vales were obtained by fitting currents recorded from each individual cell. τ1 and τ2 refer to the fast and slow inactivation time constants, respectively. Vertical bars represent S.E.M. * denotes *p* < 0.05, compared to the controls. SEP and SEN represent myocytes isolated from subepicardial and subendocardial myocardium, respectively.

We have recently reported a transmural gradient of I_Ca_ density in the normal and HF LV, with a smaller I_Ca_ density in the subendocardial (SEN) than in the subepicardial (SEP) myocytes [6]. We also demonstrated a proportional increase in I_Ca_ density and a slowed inactivation in HF LV which is caused by the excessive CaMKII-dependent phosphorylation of LTCC. Consistent with our recent findings that Kv4.3 expression can selectively inhibit membrane associated CaMKII activation by binding to the Calmodulin-binding site [7], here, we found that Kv4.3 expression reversed I_Ca_ remodeling in HF LV by reducing I_Ca_ density and acceleration of I_Ca_ inactivation, while the transmural gradient was preserved. For instance, peak I_Ca_ (recorded at test potential of +10 mV) was reduced from 7.48 ± 0.31 pA/pF (n=23) to 6.34 ± 0.35 pA/pF (n=15) for SEP and from 6.51 ± 0.25 pA/pF (n=23) to 4.9 ± 0.24 pA/pF (n=15) for SEN cells, respectively (*p* < 0.05)(Figure 3A). Interestingly, Kv4.3 expression reduced I_Ca_ density in HF ventricular myocytes to a level close to that we previously reported in the sham mouse ventricular myocytes [6].

In addition to the proportionately reduction in I_Ca_ density by Kv4.3 expression, we also evaluated the kinetics of I_Ca_ inactivation (current decay). We observed that I_Ca_ inactivation was significantly accelerated in HF myocytes transfected with Kv4.3. To quantify these changes, I_Ca_ inactivation was modeled as an exponential decay and fit by 2 exponential functions [11]. The mean values of fast (τ1) and slow (τ2) time constants in Ad-Kv4.3 transfected (n=18) and untransfected HF myocytes (n=19) were shown in Figure 3B. I_Ca_ inactivation was significantly accelerated at potentials of +10mV and above in Kv4.3 transfected HF myocytes. For example, at +10 mV, τ1 and τ2 for the Kv4.3 transfected myocytes (n=36) were 8.9 ± 0.7 ms and 67.8 ± 1.9 ms respectively, compared with 10.2 ± 0.4 ms and 74.9 ± 1.7 ms for untransfected myocytes (n=46, *p* < 0.05).

### Voltage-dependence of I_Ca_ activation and inactivation

**Figure 4:**
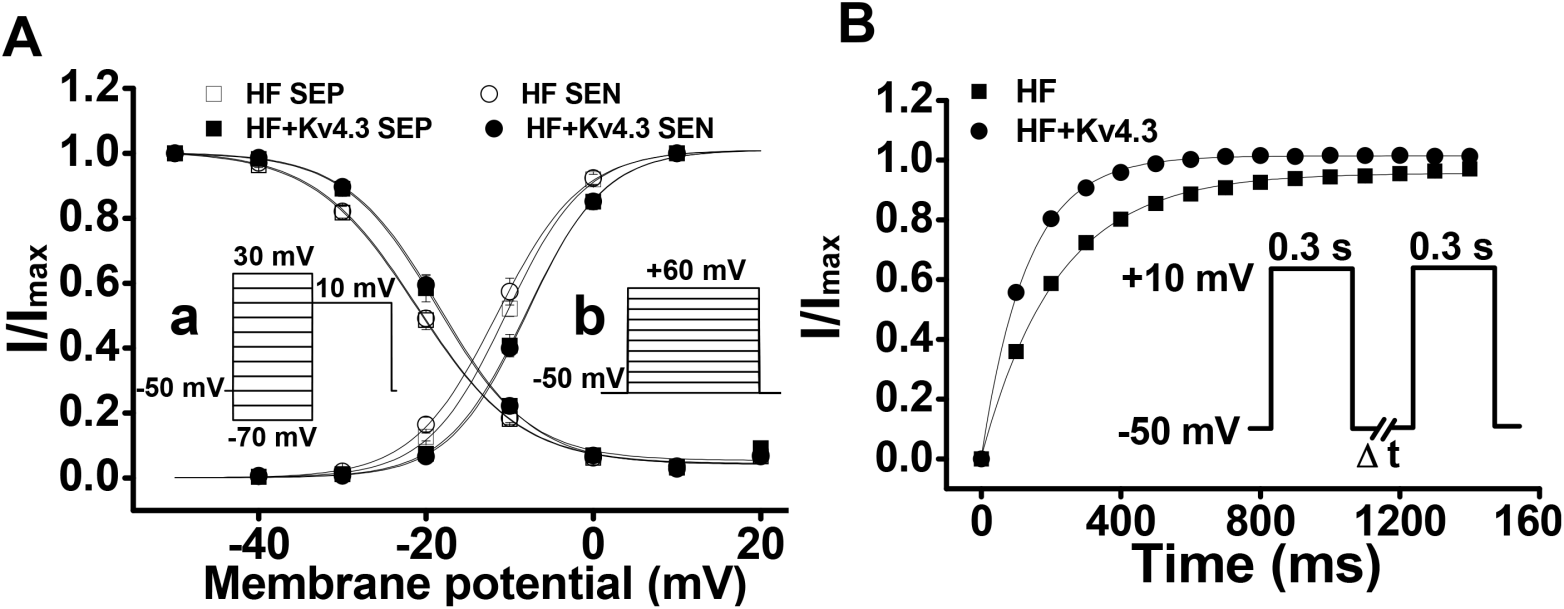
**(Panel A)** Voltage-dependent activation and inactivation of I_Ca_ recorded in the HF LV myocytes with (n=18 for SEP, n=13 for SEN) and without Ad-Kv4.3 (n=23 for both SEP and SEN) transfection, respectively. Currents elicited by each test pulse were normalized to the maximum current. The mean values are plotted versus conditioning pulse potentials (for inactivation, inset a) or test pulse potentials (for activation, inset b). Vertical bars represent S.E.M. **(Panel B)** Mean values of I_Ca_ recovery from inactivation. Paired 300 ms pulses at +10 mV were applied from a holding potential of -50 mV with the time interval (ΔT) varied from 50 ms to 1500 ms in 50 ms steps (insert). The protocol was repeated every 10 s. The mean recovery time constants were 128.2 ± 1.5 ms (n=28) for HF myocytes transfected with Ad-Kv4.3 and 216.3 ± 3.9 ms (n=31) for untransfected HF myocytes, respectively.

To measure the voltage dependence of I_Ca_ inactivation, we used a two-pulse protocol with a 300 ms conditioning pulse at potentials ranging from -70 mV to +30 mV (from holding potential of –50 mV), followed by a 300 ms test pulse to +10 mV. To determine the voltage dependence of I_Ca_ activation, we used a holding potential of –50 mV and steps of 300 ms duration test pulses from –40 to +60 in 10 mV steps. These protocols have been described in detail in our previous studies [7]. For both activation and inactivation of I_Ca_, no significant difference in voltage-dependence has been observed between SEP and SEN myocytes and between the Ad-Kv4.3 transfected and untransfected HF LV myocytes (Figure 4A).

### Accelerated I_Ca_ recovery from inactivation in HF myocytes transfected with Kv4.3

To record the time course of I_Ca_ recovery from inactivation, we held cells at a holding potential of –50 mV and applied a 300 ms pulse to +10 mV, followed by a variable time period marked as DT at –50 mV and a test pulse of 300 ms to +10 mV (insert of Figure 4B). The time constants of recovery from inactivation were evaluated by fitting data for each cell with a mono-exponential equation. In HF myocytes transfected with Kv4.3, the recovery time course was significantly accelerated (Figure 4B). The mean recovery time constants were 128.2 ± 1.5 ms (n=28) for HF myocytes transfected with Ad-Kv4.3 and 216.3 ± 3.9 ms (n=31) for untransfected HF myocytes, respectively (p<0.05). These data demonstrated that Kv4.3 expression reversed the HF-related delay of I_Ca_ recovery from inactivation to a level we reported in ventricular myocytes isolated from the normal mice [7].

### Kv4.3 expression recovered the Ca^2+^-induced I_Ca_ facilitation in HF LV myocytes

**Figure 5:**
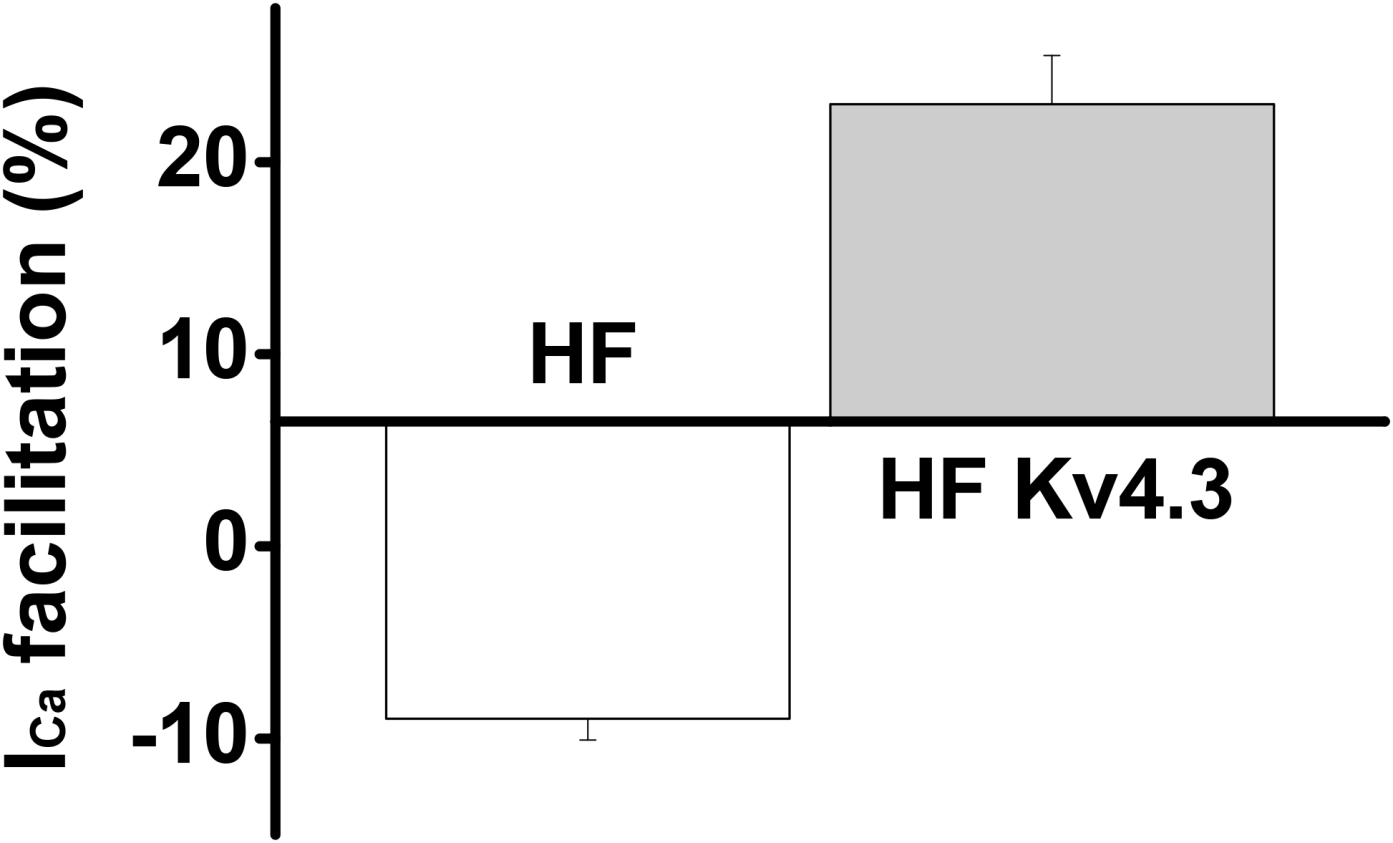
Mean values of the frequency-dependent Ca^2+^-induced I_Ca_ facilitation in HF LV myocytes with (n=39) and without Ad-Kv4.3 (n=32) transfection, respectively, showing a recovery of I_Ca_ facilitation in HF LV myocytes with Kv4.3 expression

In addition to the Increased peak I_Ca_, slowed inactivation and recovery from inactivation, another hallmark change of I_Ca_ in the HF myocytes is the blunted frequency-dependent Ca^2+^-induced I_Ca_ facilitation [6]. Here, we measured I_Ca_ facilitation in HF LV myocytes that have been transfected with Ad-Kv4.3. Consistent with our previous results recorded in HF ventricular myocytes [6], Ca^2+^-induced I_Ca_ facilitation was blunted and a frequency-dependent I_Ca_ suppression was recorded in all of the untransfected HF LV myocytes (n=32). However, Ca^2+^-induced I_Ca_ facilitation was observed in each recorded HF LV myocytes transfected with Ad-Kv4.3 (n=39) (Figure 5). These data, then, are consistent with the reduced CaMKII activity and a consequent reversion of I_Ca_ facilitation.

## DISCUSSION

There is growing body of evidence suggesting that CaMKII is an important sensor for the altered Ca^2+^ handling in HF myocytes. We have reported that in pressure-overload HF mouse LV myocytes, CaMKII activity increased by ≈2.6 fold [6]. Accordingly, I_Ca_ was significantly potentiated with increased current density and slowed inactivation (*i.e*. changing LTCC gating to mode 2) [6]. These changes facilitate Ca^2+^ influx during diastole. On the other hand, excessive CaMKII activation induces diastolic SR Ca^2+^ leak, which further increases diastolic Ca^2+^, especially in the LTCC-SR microdomain, causing a persistent CaMKII activation. The excessive activation of CaMKII in turn hyperphosphorylates LTCC, causing further remodeling of I_Ca_. Meanwhile, CaMKII-dependent RyR hyperphosphorylation causes SR Ca^2+^ leak and increase in the diastolic Ca^2+^ level, which facilitates the Ca^2+^ efflux function of sodium-calcium exchanger (I_NCX_), which, in combination with altered LTCC gating, leads to prolongation of APD and triggers abnormal impulses [14]. Therefore, I_Ca_ remodeling is a result of increased CaMKII activation and also a mediator for the disrupted calcium homeostasis in HF. Correction of I_Ca_ remodeling is an important measure contributing to normalization of calcium handling in the failing heart.

Our recent work demonstrated that Kv4.3 is an intrinsic CaMKII inhibitor which inhibits predominantly the membrane-associated CaMKII with less inhibition of the overall CaMKII [7]. In the setting of HF, Kv4.3 down-regulation directly contributes to the excessive activation of CaMKII in the membrane LTCC microdomian, causing HF-related I_Ca_ remodeling. This has been further demonstrated by a most recent study which showed that an increase in the open probability of LTCC is linked to enhanced CaMKII-mediated phosphorylation of LTCC in the membrane microdomains, contributing to the development of early afterdepolarizations [15]. Taken together, the down-regulation of Kv4.3 is an important mechanism underlying membrane-associated CaMKII activation and CaMKII-mediated I_Ca_ remodeling in HF. The main innovation of this study is to determine whether reestablishing Kv4.3 expression can effectively reverse I_Ca_ remodeling in HF.

In this study, we report a significant reduction in I_Ca_ density in the HF LV myocytes transfected with Ad-Kv4.3, leaving the transmural gradient intact. Interestingly, the mean level of I_Ca_ density recorded in the HF LV myocytes with Kv4.3 expression was quite close to the value recorded in the sham mouse LV myocytes [6]. In addition to the current density, Kv4.3 expression induced a regression of the biophysical properties of I_Ca_ in HF myocytes, *i.e.* the time courses of I_Ca_ inactivation and recovery from inactivation were accelerated. Furthermore, the frequency-dependent Ca^2+^-induced I_Ca_ facilitation which is known mediated by beat-to-beat activation of CaMKII [16], a common feature of ventricular myocytes but has been blunted in HF [^6]^, was recovered by Kv4.3 expression. Altogether, these results suggest that the expression of Kv4.3 can successfully reverse I_Ca_ remodeling in ventricular myocytes of HF.

Although CaMKII is proposed to be a novel therapeutic target for HF and HF-related ventricular arrhythmias [17-21], due to the ubiquitous distribution of CaMKII and lack of cardiac specific CaMKII inhibitor, the pharmacological CaMKII inhibition is actually not clinically applicable. To selectively inhibit the compartmentalized CaMKII activity, we propose to re-establish the intrinsic CaMKII-Kv4.3 units by *in vivo* expression of Kv4.3 in HF ventricular myocytes. Due to the membrane distribution [7], CaMKII-Kv4.3 units prevent Ca^2+^-induced CaMKII activation (*i.e*. the Ca^2+^ entered through LTCC and the Ca^2+^ released from RyR at each heart beat) and thus largely inhibit CaMKII activity in the LTCC-SR microdomain with less inhibition to the overall CaMKII. Our results revealed this pattern of CaMKII inhibition in Kv4.3 transfected HF myocytes, demonstrated by a greater reduction in p-Thr17-PLB (local CaMKII activity) than p-CaMKII (overall bulk CaMKII activity). It is predictable that restoration of the down-regulated Kv4.3 in HF LV is of great importance in normalizing Ca^2+^ homeostasis and membrane-associated ion channel remodeling via specific inhibition of the compartmentalized CaMKII activity, thus improving E-C coupling and inhibiting ventricular arrhythmias in HF. This opens a direction of future study.

### Limitations

As an endogenous CaMKII inhibitor, Kv4.3 mainly affects CaMKII activity in the sarcolemmal compartment due to their membrane localization in myocytes. Meanwhile, Kv4.3 expression also leads to APD shortening and a consequent reduction in [Ca^2+^]_i_, which decreases the overall intracellular CaMKII activity. This is exactly what we observed in this study. However, since the LTCC is a membrane protein, the membrane-localized CaMKII activity is accordingly much more important for the regulation of I_Ca_ than the bulk CaMKII activity. In this study, we have demonstrated the key role of Kv4.3 expression in the regression of I_Ca_ remodeling in HF ventricular myocytes. We cannot, however, dissect the contribution of the overall CaMKII activation which is associated with the APD prolongation. This portion of contribution (if any) would be minor due to the fact of small change in the bulk CaMKII activity which is also away from the LTCC location. As an advantage, we recorded I_Ca_ in the myocytes with and without successful Kv4.3 expression in the same heart, which avoided the influence of heart condition and the variations of the dissociation-related cell quality.

In addition to I_Ca_, other membrane currents, such as I_Na_, I_k1_ and I_NCX_ may also be modified by Kv4.3 expression since they are direct substrates of CaMKII. This may lead to a complex change in the cellular Ca^2+^ homeostasis and electrical property. These are certainly the interest of future study.

## MATERIALS AND METHODS

All the animal experiments were performed conforming the NIH guidelines (Guide for the care and use of laboratory animals) with the protocols approved by the institutional Animal Care and Use Committee.

### Severe thoracic aortic banding

Increased pressure in the proximal aorta was induced by severe thoracic aortic banding (sTAB) [6, 22]. Briefly, male C57BL6 mice (6-8 weeks old) were anesthetized with ketamine (100 mg/kg IP) plus xylazine (5 mg/kg IP). The mice were then ventilated at 120 breaths per minute at 0.1 mL tidal volume. A 3-mm left-sided thoracotomy was created at the second intercostal space. The transverse aortic arch was ligated (7-0 Prolene) between the innominate and left common carotid arteries with an overlying 28-gauge needle. The needle was then removed, leaving a discrete region of stenosis. The chest was closed using tissue adhesive.

### Isolation of individual ventricular myocytes

Mice were euthanized with pentobarbital (150 mg/kg *IP*) and hearts were then removed through open chest surgery. LV myocytes were isolated enzymatically by a protocol we described previously [6]. In brief, after retrograde perfusion with Krebs-Ringer solution (2 mL/min for 5 min), the heart was perfused by a fresh solution containing 1 mg/mL Worthington type II collagenase for another 15 - 20 minutes. The heart was then removed and the LV wall was cut and put into a culture dish filled with “KB” solution. A small 90-degree curved forceps was used to carefully dissect a very thin layer of endomyocardium. The mid-region myocardium was dissected and discarded, leaving the remainder as epimyocardium. Endomyocardium and epimyocardium were minced into small pieces in “KB” solution, triturated and filtered. Only calcium-tolerant, quiescent and rod-shaped cells showing clear cross striations were studied.

### *In vivo* Kv4.3 gene transfection

Multi-site adenovirus injection was performed through a 1 cm incision in the left side sixth intercostal space in HF mice. Delivery of adenoviral vectors into the LV myocardium can be performed safely under visual control. In order to increase the transfection rate and reduce the toxicity, adenoviruses Ad-Kv4.3 was purified before use [7]. A total volume of 12 μL adenovirus at 1.6 x 10^12^ plaque forming unit was distributed into the LV apex, anterior, lateral, and posterior walls with two injections for each region and 1.5 μl for each injection. 7 days after injection, mice were used for myocytes isolation.

### Electrophysiological recordings

Currents were recorded in the LV myocytes isolated from HF mice 7 days after *in vivo* LV myocardium injection with Ad-Kv4.3. GFP positive myocytes were selected for I_Ca_ and outward K^+^ current recording under fluorescence microscope (Ad-K4.3 contains GFP reporter gene), while the currents in the GFP negative myocytes were recorded as control. Because I_Ca_ has transmural gradient in HF LV [^6]^, we have separated the subepicardial and subendocardial myocytes for data acquisition and analysis.

I_Ca_ and outward K^+^ current were recorded by whole-cell voltage clamp technique, with pipette resistances of 2-3 M when filled with internal solution. I_Ca_ was recorded at room temperature. Cells were depolarized every 10 s from a holding potential of -50 mV to test potentials between -40 to +60 mV (10mV steps) for 300 ms. To minimize the impact of I_Ca_ run-down, we added 5 mmol/L Mg-ATP to the pipette solution and conducted data acquisition after 5 - 10 minutes of equilibration between pipette solution and intracellular contents [6]. Cells showing continuous current run-down were excluded from the analysis. For recording of outward K^+^ current, CdCl_2_ (0.2 mM) was used to block Ca^2+^ currents and the calcium-dependent transient outward current (I_to2_) [6]. Myocytes were depolarized for 5000 ms from a holding potential of -80 mV to potentials from -40 mV to +60 mV in 10 mV voltage steps at a pulse interval of 15s. I_Na_ was eliminated by applying a 30 ms pre-pulse from holding potential to -40 mV.

### Solutions

The Krebs-Ringer solution for cell isolation contained (mmol/L): NaCl 35, KCl 4.75, KH_2_P0_4_ 1.19, Na_2_HP0_4_ 16, Sucrose 134, NaCO_3_ 25, Glucose 10, HEPES 10, pH 7.4 with NaOH. The “KB” solution for storage of cells contained (mmol/L): taurine 10, glutamic acid 70, KCl 25, KH_2_PO_4_ 10, glucose 22, EGTA 0.5, pH adjusted to 7.2 with KOH. Perfusion solution for I_Ca_ recording contained (mmol/L): TEA 135, MgCl_2_ 0.53, CaCl_2_ 1.8, CsCl 20, HEPES 5. pH 7.4 with CsOH. Pipette solution for I_Ca_ recording contained (mmol/L): CsOH 110, aspartic acid 90, CsCl 20, tetraethylammonium Cl (TEA-Cl) 10, HEPES 10, EGTA 10, Mg-ATP 5, Na_2_ creatine phosphate 5, GTP (Tris) 0.4, leupeptin 0.1, pH 7.2 with CsOH. Tyrode’s solution for outward K^+^ current recording (mM): NaCl 135, MgCl_2_ 1.1, CaCl_2_ 1.8, KCl 5.4, HEPES 10, glucose 10, pH 7.4 with NaOH. Pipette solution for outward K^+^ current recordings (mM): KCl 130, MgCl_2_ 1, HEPES 10, EGTA 5, Mg-ATP 5, Na_2_-creatine phosphate 5, pH 7.2 with KOH.

### Western blot analysis

Myocytes were homogenized in 1% TX-100 buffer [50 mM Tris-HCl, pH 7.4, 4% glycerol, 1 mM DTT, 1% Triton X-100, 1 mM EDTA, Mini-Complete^®^ protease inhibitor cocktail (Roche), and phosphatase inhibitor cocktails I and II (Sigma-Aldrich)] and immunoblotted for Kv4.3, CaMKII, phospho-CaMKII (p-CaMKII), p-PLB-Ser16, Phospho-Thr17-PLB, PLB, and GAPDH, respectively. Primary antibodies used in this study include rabbit polyclonal and mouse monoclonal anti-CaMKII antibodies (M-176 and G-1) (Santa Cruz Biotechnology, Santa Cruz, CA), polyclonal anti-phospho-CaMKII (Thr287) antibody (Cell Signaling solutions, Lake Placid, NY), goat polyclonal anti-PLB antibody (Santa Cruz Biotechnology), rabbit polyclonal anti-P-Ser16PLB antibodies (Badrilla, Leeds, UK), rabbit polyclonal anti-P-Thr17PLB antibodies (Badrilla, Leeds, UK), rabbit polyclonal antibody against Kv4.3 (Alomone Labs, Jerusalem, Isarael) and mouse monoclonal anti-GAPDH antibody (Millipore Corporation).

### Statistical analysis

I_Ca_ amplitude was evaluated as the peak inward current. Biexponential functions were used to model current decay kinetics. Peak outward K^+^ current amplitude was measured as the difference between the peak current and the sustained current remaining at the end of a 5000 ms pulse. In order to correct for variability in cell size, current were expressed as current density by dividing the absolute current amplitude by cell capacitance (pA/pF). Results are expressed as mean ± SEM. Statistical analysis was performed using Sigmastat for Windows (Version 3.5).). The paired and unpaired *t*-test was used for the comparisons and *p*<0.05 was regarded as significant.
